# Editorial: Biodegradation of agricultural pesticides

**DOI:** 10.3389/fmicb.2026.1874629

**Published:** 2026-05-28

**Authors:** Bijay Kumar Behera, Wenjie Ren, Ashwani Kumar

**Affiliations:** 1 National Fisheries Development Board (NFDB), Department of Fisheries, Government of India, Hyderabad, Telangana, India; 2 Aquatic Environmental Biotechnology Division, ICAR-Central Inland Fisheries Research Institute, Barrackpore, West Bengal, India; 3 State Key Laboratory of Soil and Sustainable Agriculture, Institute of Soil Science, Chinese Academy of Sciences, Nanjing, China; 4 Department of Agricultural resources and Environment, University of Chinese Academy of Sciences, Nanjing, China; 5 Metagenomics and Secretomics Research Laboratory, Department of Botany, University of Allahabad (A Central University), Prayagraj, Uttar Pradesh, India

**Keywords:** agricultural pesticides, environmental microbiology, microbial biodegradation, microbial consortia, soil health, sustainable agriculture

The extensive use of agricultural pesticides has substantially enhanced crop yields and food security. However, the persistent presence of pesticide residues in soil and water has emerged as a significant environmental and public health concern. Numerous conventional pesticides exhibit chemical stability, long half-life, and resistance to natural degradation, leading to toxic accumulation within ecosystems. This persistence not only affects soil microbial populations and undermines ecological equilibrium but also endangers non-target species, including humans, through bioaccumulation and translocation within the food chain (Zhou et al., 2025; Pathak et al., 2022).

Conventional techniques for pesticide elimination predominantly rely on physicochemical processes such as hydrolysis, photolysis, and chemical oxidation. While these methods may reduce pesticide levels, they possess considerable limitations. For example, chemical degradation often generates detrimental byproducts, consumes substantial energy, and incurs high operational expenses. Moreover, these approaches frequently fail to fully decompose complex pesticide compounds, underscoring the need for alternative, sustainable solutions (Mitra et al., 2026; Bhargav et al., 2026).

In this context, microbial-mediated biodegradation has emerged as a viable, ecologically sustainable approach for mitigating pesticide pollution. Microorganisms, including bacteria, fungi, and actinomycetes, exhibit exceptional metabolic plasticity, enabling them to use pesticides as carbon or energy sources. These microbes may enzymatically convert complex and hazardous pesticide compounds into simpler, less toxic substances. Under favorable conditions, they can even achieve complete mineralization, breaking down pesticides into water, carbon dioxide, and inorganic components (Srivastava et al., 2026; Singh and Kumawat, 2025).

The accuracy and effectiveness of microbial degradation are primarily governed by the enzymatic systems encoded in microbial genomes. Essential enzymes, including hydrolases, oxygenases, dehydrogenases, and transferases, are pivotal in the degradation of pesticides via hydrolysis, oxidation, reduction, and dehalogenation. The considerable variation in microbial metabolic pathways underscores the importance of investigating and characterizing microbial strains to evaluate their degradative potential. Comparative investigations of these strains yield significant insights into their degradation efficiency, substrate selectivity, and adaptability to diverse environmental conditions (Srivastava et al., 2026; Dey et al., 2025).

Advancements in biological sciences and omics technologies have considerably enhanced our comprehension of microbial biodegradation pathways. Metagenomics, transcriptomics, and proteomics have been utilized to elucidate functional genes, regulatory networks, and metabolic processes associated with pesticide degradation. These methods have enabled the identification of new microbial taxa and enzymes capable of degrading resistant pesticides. Furthermore, they have enabled researchers to examine microbial communities within their natural environments, yielding significant insights into *in situ* biodegradation (Chandran et al., 2020; Shahid, 2025).

Another notable innovation is the application of genetic and metabolic engineering to enhance microbial degrading abilities. By manipulating specific genes and metabolic pathways, researchers can improve the efficiency, accuracy, and stability of microbial strains for pesticide breakdown. Engineered microbes can be customized to degrade specific classes of pesticides, achieve rapid degradation rates, and operate optimally across various environmental conditions. Genetic engineering enables the formation of microbial consortia with complementary metabolic functions, thereby enabling synergistic degradation of intricate pesticide mixtures (Olawade et al., 2025; Jaiswal et al., 2019).

Researchers are developing genetically modified microorganisms (GMMs) with improved biodegradation capabilities for the practical remediation of pesticide-contaminated environments. GMMs can be introduced into polluted soils and aquatic environments to accelerate degradation and detoxification. However, their application raises concerns about biosafety, potential threats to indigenous ecosystems, and regulatory challenges. Careful risk evaluation and appropriate use of genetic engineering methods are essential for solving these issues (Misra et al., 2024).

Beyond pesticide removal, microbial biodegradation can also positively affect soil quality and crop production. Soil health is predicated on living soil, where microbe populations help to drive nutrient cycling, decompose organic matter, and support plant growth. When these processes are disrupted by pesticide residues, soil quality and crop productivity are diminished. Microbial biodegradation not only eliminates such residues but also revitalizes beneficial soil microbes. Furthermore, certain degradative bacteria can promote plant growth and augment crop yield (Bertola et al., 2021).

The successful *in situ* application of microbial biodegradation requires careful consideration of environmental factors such as soil composition, temperature, moisture, pH, and the presence of other contaminants. Strategies implemented at the field scale may involve bioaugmentation, adding specialized microbial strains to the environment or biostimulation approaches that enrich the native microbial population by adding nutrients or soil amendments. The efficacy of these strategies depends on several factors, including the adaptability and survival of introduced or enriched microbial strains, as well as their interactions with native microbes (Kuppan et al., 2024).

**Figure 1:**
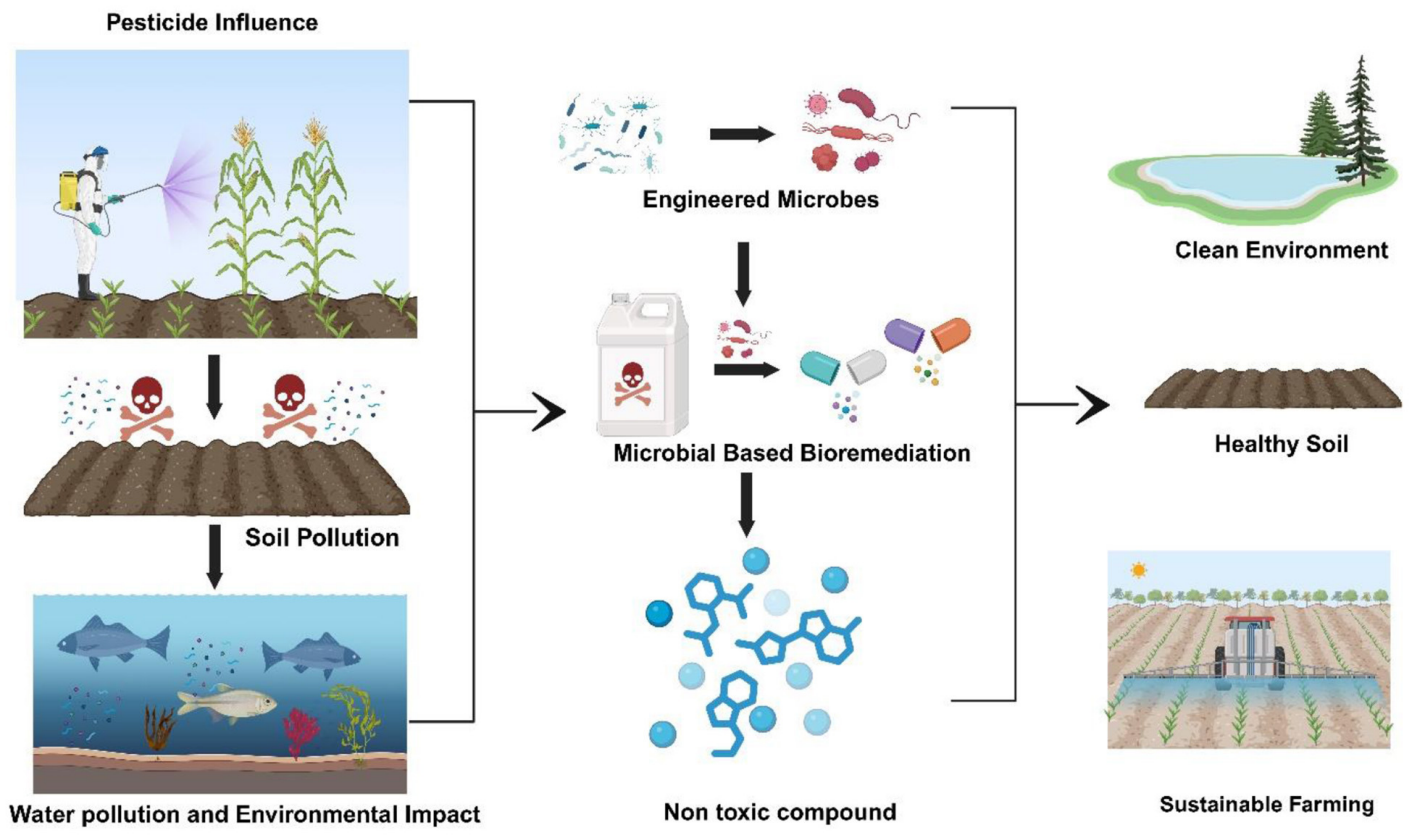
Provides a comprehensive examination of microbial biodegradation principles for agricultural pesticides, emphasizing the transition from environmental pollution to ecological restoration through both natural and synthetic microbial techniques.

This Research Topic aims to compile cutting-edge research and reviews that shed light on current knowledge of microbial biodegradation of pesticide. Contributions range from the isolation and characterization of novel microbial strains with pesticide-degrading abilities to comparative evaluations of degradation efficiency across various strains. The metabolic pathways and mechanisms used for transformation are described. The impacts of biodegradation on agricultural productivity and soil quality are highlighted. Advancement in microbial engineering constitutes another important theme of this Research Topic. A further major theme concerns the engineering of microbial systems: studies focusing on synthetic biology, gene editing, and metabolic pathway manipulation highlight the potential of engineered microbes for pesticide cleanup. Finally, innovative methodologies, including novel analytical instruments, experimental frameworks, and biotechnological applications, are presented to establish core procedures that will guide further research in this discipline (Srivastava et al., 2026) (Figure 1).

Sustainable agriculture is a crucial component of this Research Topic. Microbial applications support resilient, ecologically sound agricultural practices by reducing the necessity for chemical treatments and mitigating the adverse environmental impacts of pesticides. In doing so, they align with global initiatives aimed at promoting sustainable agriculture, safeguarding ecosystem health, addressing food security and align with sustainable development goals (SDGs) of the United Nations (Kuppan et al., 2024).

Despite the advancements documented in this Research Topic, several challenges continue to hinder the widespread application of microbial biodegradation of pesticides. The intricacy of pesticide mixtures, variable environmental factors, and our still-limited understanding of microbial communities all constrain the field-scale application of these techniques. Furthermore, regulatory concerns regarding the environmental release of genetically modified organisms must be carefully addressed to ensure safety and secure public acceptance. Overcoming these obstacles will require close collaboration among experts in microbiology, environmental science, biotechnology, agriculture, and regulatory policy (Kumar et al., 2025).

Further studies should focus on developing resilient, sustainable biodegradation techniques tailored to field conditions. Promising strategies include constructing more stable and functional microbial consortia, engineering carrier materials for effective microbial delivery, and optimizing field application techniques. Innovations in digital farming and precision agriculture offer new opportunities to monitor and manage biodegradation processes, enabling more precise and effective interventions (Kuppan et al., 2024).

In conclusion, microbial biodegradation offers a viable and increasingly effective approach to mitigating the environmental impacts of pesticide use in agriculture. By harnessing and enhancing microorganisms' inherent metabolic capabilities with modern biotechnological tools, the degradation of pesticide residues can be made effective, cost-efficient and environmentally sustainable. This Research Topic features articles that emphasize the role of microbial systems in advancing sustainable agriculture (Srivastava et al., 2026). This Research Topic aims to promote future research, foster interdisciplinary collaborations, and drive innovations in microbial biotechnology. We can facilitate the transition toward a productive and sustainable agricultural system by connecting scientific discovery with practical applications.
